# Risk factors for major depressive disorder among adults recruited from hospital settings in Uganda, Kenya, Ethiopia and South Africa

**DOI:** 10.1038/s41467-026-77189-6

**Published:** 2026-08-27

**Authors:** Allan Kalungi, Wilber Ssembajjwe, Kester B. B. Tindi, Emmanuel K. Mwesiga, Jared G. Maina, Fred Kirumira, Elizabeth G. Atkinson, Lukoye Atwoli, Mark Barker, Lori B. Chibnik, Symon M. Kariuki, Charles R. J. C. Newton, Rocky E. Stroud, Solomon Teferra, Zukiswa Zingela, Alicia R. Martin, Moffat Nyirenda, Dan J. Stein, Eugene Kinyanda, Karoline Kuchenbaecker, Segun Fatumo, Dickens H. Akena

**Affiliations:** 1 Department of Non-communicable Disease Epidemiology (NCDE), London School of Hygiene and Tropical Medicine, London, UK; 2 Medical Research Council/Uganda Virus Research Institute and London School of Hygiene and Tropical Medicine (MRC/UVRI & LSHTM) Uganda Research Unit, Entebbe, Uganda; 3 Department of Medical Biochemistry, School of Biomedical Sciences, College of Health Sciences, Makerere University, Kampala, Uganda; 4 School of Psychology and Vision Sciences, University of Leicester, Leicester, UK; 5 Department of Psychiatry, School of Medicine, College of Health Sciences, Makerere University, Kampala, Uganda; 6 Social, Genetic and Developmental Psychiatry Centre, Institute of Psychiatry, Psychology & Neuroscience, King’s College London, de Crespigny Park, London, SE5 8AF UK; 7 Department of Molecular and Human Genetics, Baylor College of Medicine, Houston, Texas USA; 8 Department of Mental Health, School of Medicine, Moi University College of Health Sciences, Eldoret, Kenya; 9 Brain and Mind Institute and Department of Internal Medicine, Medical College East Africa, the Aga Khan University, Nairobi, Kenya; 10 Stanley Center for Psychiatric Research, Broad Institute of MIT and Harvard, Cambridge, MA USA; 11 Department of Neurology, Massachusetts General Hospital, Boston, MA USA; 12 Neurosciences Unit, Clinical Department, KEMRI-Wellcome Trust Research Programme-Coast, Kilifi, Kenya; 13 Department of Psychiatry, University of Oxford, Oxford, UK; 14 Research Division, African Population and Health Research Center, Nairobi, Kenya; 15 Department of Epidemiology, Harvard T.H. Chan School of Public Health, 677 Huntington Ave Room 505F, Boston, MA 02115 USA; 16 Department of Psychiatry, School of Medicine, College of Health Sciences, Addis Ababa University, Addis Ababa, Ethiopia; 17 Executive Dean’s Office, Faculty of Health Sciences, Nelson Mandela University, Port Elizabeth, South Africa; 18 Analytic and Translational Genetics Unit, Massachusetts General Hospital, Boston, MA USA; 19 Program in Medical and Population Genetics, Broad Institute of Harvard and MIT, Cambridge, MA USA; 20 South African Medical Research Council (SAMRC) Unit on Risk and Resilience in Mental Disorders, Department of Psychiatry and Neuroscience Institute, University of Cape Town, Cape Town, South Africa; 21 Division of Psychiatry, University College London, London, UK; 22 Precision Healthcare University Research Institute, Queen Mary University of London, London, UK

**Keywords:** Risk factors, Depression

## Abstract

Major depressive disorder (MDD) significantly contributes to the mental health burden in Africa, requiring insights into prevalence and risk factors to inform culturally responsive care. This study assessed current and lifetime MDD prevalence and associated factors among 7,073 adults from hospital settings in Uganda, Kenya, Ethiopia, and South Africa within the NeuroGAP-Psychosis study. Prevalence was 0.9% for current MDD and 5.1% for lifetime MDD, substantially lower than prior estimates from the region. Lower rates may be due to the population studied or cultural variations in expression of symptoms. Multilevel logistic regression identified significant associations between current MDD and negative life events, alcohol use, and Kessler psychological distress scores, while lifetime MDD was linked to age, female sex, chronic pain, frequent headaches, and positive psychosis screening. These findings underscore the need for targeted mental health interventions tailored to the identified factors to reduce the burden of MDD in diverse African contexts.

## Abstract (web summary)

Across Uganda, Kenya, Ethiopia and South Africa, a harmonized study identified factors associated with major depression and found country differences explained 24% of variation, indicating regional strategies should be adapted to national contexts.

## Introduction

Major depressive disorder (MDD) is a common mental disorder characterized by persistent feelings of sadness, hopelessness, and lack of interest in normally pleasurable activities^1^.

The global prevalence of MDD has been estimated at 3.4%, accounting for point, 12-month, and lifetime prevalence using pooled prevalence ratios^2^. It is estimated that 10 − 15% of the general population experience clinical depression in their lifetimes with 5% of men and 9% of women experiencing a depressive disorder in a given year^3^. According to the World Health Organisation (WHO), MDD was responsible for over 50 million years lived with disability (YLD) in 2015 and can lead to suicide which is responsible for up to 800,000 deaths annually^4^. MDD leads to high levels of morbidity and a 10% increased risk of all-cause mortality^5^.

The burden of MDD has been reported to vary by the WHO region and the study population. It has been reported to vary from 3.6% in the Western Pacific region to 5.4% in the African region^4^. Africa accounts for over 9% of the global burden of MDD^4^, yet it is a region where very low treatment rates have been reported for MDD^6,7^.

From existing limited data, in Sub-Saharan Africa (SSA) an age-standardized prevalence of 4.5% has been estimated for MDD^2^. The age-standardized prevalence of MDD has been reported to be higher in SSA than elsewhere globally^2^. In South Africa, a prevalence of 4.6%^8^ has been reported among the general population while prevalence rates of 1.9−38% have been reported among small controlled populations. In Uganda, a pooled prevalence of 30.2% has been determined by a systematic review and a meta-analysis among heterogeneous samples^9^ while MDD prevalence estimates of 4.2–29.3% have been determined among general populations from various study sites in Uganda^10–14^. In Ethiopia, a prevalence of 4.7% has been reported among the general population, while prevalence rates of 4.8 – 57% have been reported among heterogeneous samples^15,16^. In Kenya, a prevalence of 4.4% has been reported among the general population, while prevalence rates of 6.3–72.9% have been reported among heterogeneous samples^17,18^.

Despite accumulating data on MDD prevalence across various regions in SSA, a comprehensive understanding of the disorder’s epidemiological landscape remains elusive. Reported prevalence estimates vary widely across studies and settings in the region, reflecting differences in study populations and methods employed in assessing for MDD^2,8,9,16^. Additionally, while links between MDD and lifestyle diseases such as diabetes^19^ and hypertension^20^, as well as infectious diseases like HIV, Ebola, and COVID-19^21,22^ have been noted, there is a dearth of studies exploring these relationships. The inconsistent use of diagnostic criteria and potential underdiagnosis due to cultural perceptions of mental health highlight the need for more localized and culturally adapted research methodologies. Also, given the heterogeneous nature of MDD^23^, it is important to understand the rates and risk factors for MDD across different populations to inform policies aimed at reducing the burden of the disorder. This study aimed to investigate the prevalence and specific risk factors (demographic, social and family dynamics, lifestyle and health behaviors, physical health conditions, psychological and geographical/cultural) for MDD among a cross-sectional sample of participants recruited from hospital settings from Eastern (Uganda, Kenya, Ethiopia) and Southern (South Africa) Africa.

In this work, we investigate the prevalence and risk factors for current and lifetime MDD using a harmonized, multi-country dataset from Uganda, Kenya, Ethiopia, and South Africa and a standardized Mini-International Neuropsychiatric Interview (MINI, version 7.0.2)^24^, enabling direct cross-national comparison. We show that the prevalence of both current and lifetime MDD varies across countries and is generally lower than reported in many previous studies from SSA. Using multilevel modeling, we identify demographic, behavioral, psychosocial, and clinical factors associated with MDD and quantify the extent of between-country variation. These findings provide a standardized cross-country assessment of MDD and offer evidence to inform mental health research, prevention, and service delivery in the region.

## Results

The socio-demographic characteristics and clinical variables of the study participants are shown in Table 1.

**Table 1:** Description of the socio-demographic characteristics and clinical variables of the study participants

Factor	Level	Frequency (Percentage) *n *= 7073
Sex at birth	Female	2259 (31.9%)
	Male	4814 (68.1%)
Age	Mean (SD)	37.1 (11.5)
Marital status	Married/living together	3504 (49.6%)
	Widowed/divorced/separated	822 (11.6%)
	Single	2744 (38.8%)
Current living arrangement	Lives alone	1077 (15.3%)
	Lives with family & friends	5978 (84.7%)
Highest level of education	Primary or less	1710 (24.2%)
	Secondary	3219 (45.5%)
	Tertiary	2141 (30.3%)
Study country	South Africa	1805 (25.5%)
	Kenya	682 (9.6%)
	Uganda	2283 (32.3%)
	Ethiopia	2303 (32.6%)
Current use of tobacco products	No	4803 (67.9%)
	Yes	2270 (32.1%)
Current use of alcoholic beverages	No	2593 (36.7%)
	Yes	4480 (63.3%)
Current use of substances	No	4996 (70.6%)
	Yes	2077 (29.4%)
Blood pressure	Normal	5050 (71.4%)
	High	2023 (28.6%)
Body mass index	Mean (SD)	23.9 (4.9)

*SD* standard deviation.

### Prevalence of major depressive disorder

As shown in Table 2, the overall prevalence of current and lifetime MDD from this study was 0.9 and 5.1%, respectively. For current MDD at country level, South Africa had the highest prevalence (2.1%) followed by Uganda (0.7%), Ethiopia (0.5%) and Kenya (0.1%). For lifetime MDD at the country level, South Africa still had the highest prevalence (8.4%), followed by Uganda (7.9%), Ethiopia (1.2%), and the lowest being Kenya (0.3%).

**Table 2:** Prevalence of major depressive disorder among the study participants

Study Site	Number of participants	Number of MDD cases	Prevalence (95% CI)
a) **Current major depressive disorder**			
South Africa	1805	37	2.1% (1.5, 2.8)
Uganda	2283	16	0.7% (0.4, 1.1)
Ethiopia	2303	12	0.5% (0.3, 0.9)
Kenya	682	01	0.1% (0.02, 1.0)
Combined in all 4 countries	7073	66	0.9% (0.7, 1.2)
b) **Lifetime major depressive disorder**			
South Africa	1805	151	8.4% (7.2, 9.7)
Uganda	2283	180	7.9% (6.8, 9.1)
Ethiopia	2303	27	1.2% (0.08, 1.7)
Kenya	682	2	0.3% (0.07, 1.2)
Combined in all 4 countries	7073	360	5.1% (4.6, 5.^1^6)

*MDD* major depressive disorder, *CI* confidence interval.

### Factors associated with current major depressive disorder

In the multilevel models, male sex at birth was marginally significantly associated with lower odds of current MDD (aOR = 0.47 [95% CI: 0.22; 1.02]). Level of education did not show strong or consistent associations with MDD; neither secondary nor tertiary education was significantly associated with MDD in any model. Living arrangements and marital status were similarly not associated with significant changes in the odds of MDD across the models. Several behavioral and health-related factors were significantly associated with MDD. Current use of alcohol was strongly associated with MDD (aOR = 6.07 [95% CI: 1.89; 19.46]). On the other hand, current substance use and tobacco consumption were not significantly associated with MDD. Experiencing traumatic life events was significantly associated with MDD (aOR = 2.16 [95% CI: 1.00; 4.66]). Among chronic conditions, frequent headaches were significantly associated with MDD (aOR = 1.89 [95% CI: 1.02; 3.50]), while other chronic conditions, such as arthritis and chronic back/neck pain, did not show significant associations. Higher scores on the Kessler psychological distress scale (K10) were significantly associated with MDD (aOR = 1.26 [95% CI: 1.21; 1.31]). The geographical location, represented by living in Eastern Africa (Uganda, Kenya, and Ethiopia) compared to Southern Africa (South Africa), was not significantly associated with MDD. The ICC for the fully adjusted model (model 3) was 0.14 (95% CI: 0.09; 0.32), indicating that 14% of the variance in MDD was attributable to differences between countries. These results are summarized in Table 3a.

**Table 3:** a Results of fitting a multilevel logistic regression model for factors associated with current major depressive disorder

Factor	Model 1 aOR (95% CI)	Model 2 aOR (95% CI)	Model 3 aOR (95% CI)
**Age**	1.00 (0.97; 1.02)	0.98 (0.95; 1.01)	0.98 (0.95; 1.02)
**Sex at birth**			
Female (reference)	1.00	1.00	1.00
Male	0.55 (0.31; 1.00)	0.44 (0.20; 0.94)	0.47 (0.22; 1.02)
**Highest level of education**			
Primary or less (reference)	1.00	1.00	1.00
Secondary	0.74 (0.38; 1.44)	0.71 (0.33; 1.52)	0.88 (0.40; 1.96)
Tertiary	0.99 (0.47; 2.07)	1.57 (0.66; 3.77)	1.70 (0.71; 4.09)
**Current living arrangement**			
Lives alone (reference)	1.00	1.00	1.00
Lives with family & friends	1.61 (0.74; 3.54)	2.00 (0.76; 5.22)	2.09 (0.79; 5.50)
**Marital status**			
Married/Living Together (reference)	1.00	1.00	1.00
Widowed/Divorced/Separated	2.07 (1.03; 4.12)	1.54 (0.66; 3.58)	1.61 (0.69; 3.75)
Single	1.13 (0.59; 2.15)	0.88 (0.42; 1.84)	0.97 (0.46; 2.05)
**Use of alcoholic beverages**			
No (reference)		1.00	1.00
Yes		5.51 (1.76; 17.20)	**6.07 (1.89; 19.46)**^**a**^
**Substance use**			
No (reference)		1.00	1.00
Yes		0.66 (0.32; 1.36)	0.68 (0.33; 1.40)
**Use of tobacco products**			
No (reference)		1.00	1.00
Yes		1.75 (0.80; 3.81)	2.19 (0.97; 4.95)
**Blood pressure**			
Normal (reference)		1.00	1.00
High		1.71 (0.91; 3.21)	1.89 (0.99; 3.61)
**Body mass index**		0.97 (0.92; 1.02)	0.97 (0.93; 1.03)
**Negative life events**			
No (reference)		1.00	1.00
Yes		1.90 (0.89; 4.03)	**2.16 (1.01; 4.66)**^**b**^
**Chronic conditions**			
Arthritis		0.88 (0.34; 2.25)	0.91 (0.36; 2.33)
Chronic back/neck pain		0.91 (0.45; 1.85)	0.91 (0.45; 1.85)
Frequent headaches		1.86 (1.01; 3.43)	**1.89 (1.02; 3.50)**^**c**^
Cancer		0.39 (0.04; 3.73)	0.40 (0.04; 3.76)
**Kessler Psychological Distress Score**		1.25 (1.20; 1.30)	**1.26 (1.21; 1.31)**^**d**^
**Psychosis screening**			
Past year		0.88 (0.31; 2.45)	0.89 (0.32; 2.49)
Lifetime		1.91 (0.80; 4.55)	1.97 (0.82; 4.72)
**Country geographical location**			
Southern Africa (reference)			1.00
Eastern Africa			2.12 (0.88; 5.09)
**ICC**	0.18 (0.03; 0.59)	0.15 (0.09; 0.36)	0.14 (0.09; 0.32)

*CI* confidence intervals, *aOR* adjusted odds ratios, *ICC* intraclass correlation coefficient.

* Multilevel (random-intercept) logistic regression; effect sizes are aOR with 95% CI, and ICC (95% CI) reflects between-country variance. All hypothesis tests are two-sided Wald z-tests (asymptotic; no degrees of freedom apply); no adjustment was made for multiple comparisons.

Superscripts denote exact two-sided Model 3 *P* values: ^a^*P *= 0.002; ^b^*P* = 0.050; ^c^*P* = 0.043; ^d^*P* < 0.001.

### Factors associated with lifetime major depressive disorder

In the multilevel models, age and sex were significant predictors of lifetime MDD. Increased age was negatively associated with MDD (adjusted odds ratio (aOR) of 0.98 [95% CI: 0.97; 0.99]) in the fully adjusted model). Males had significantly lower lifetime MDD compared to females (OR of 0.69 [95% CI: 0.50; 0.94]). Education, living arrangements, and marital status were not significantly associated with MDD. Several behavioral and health-related factors were also significantly associated with MDD. Alcohol consumption was also associated with MDD (aOR of 1.41 [95% CI: 1.03; 1.93]), while substance and tobacco use were not significantly associated with MDD. Experiencing negative life events was strongly associated with MDD, with those reporting such events having more than twice the odds (aOR = 2.32, [95% CI: 1.72; 3.13]) compared to those who did not. Among chronic conditions, chronic back/neck pain (aOR = 1.53 [95% CI: 1.15; 2.04]) and frequent headaches (aOR = 1.63 [95% CI: 1.25; 2.12]) were significantly associated with MDD, while other chronic conditions like arthritis and cancer were not. Higher scores on the K10 were also associated with MDD (aOR = 1.17 [95% CI: 1.14; 1.19]). Additionally, psychosis screening results showed that both past-year psychosis experiences (aOR = 0.49 [95% CI: 0.30; 0.79]), and lifetime psychosis experiences were associated with MDD (aOR = 2.92 [95% CI: 2.11; 4.03]). Geographical location was not a significant factor, as odds of MDD did not statistically differ between individuals from Eastern Africa and those from Southern Africa. The ICC for the fully adjusted model was 0.24 [95% CI: 0.05; 0.64], indicating that 24% of the variance in MDD was attributable to differences between countries. These results are summarized in Table 3b.

**Table 3:** b Results of fitting a multilevel logistic regression model for factors associated with lifetime major depressive disorder

Factor	Model 1 aOR (95% CI)	Model 2 aOR (95% CI)	Model 3 aOR (95% CI)
**Age**	0.99 (0.98; 1.00)	0.98 (0.97; 0.99)	**0.98 (0.97; 0.99)**^**a**^
**Sex at birth**			
Female (reference)	1.00	1.00	1.00
Male	0.66 (0.51; 0.55)	0.69 (0.50; 0.95)	**0.69 (0.50; 0.94)**^**b**^
**Highest level of education**			
Primary or less (reference)	1.00	1.00	1.00
Secondary	0.84 (0.63; 1.13)	1.00 (0.73; 1.38)	1.00 (0.72; 1.38)
Tertiary	1.17 (0.84; 1.62)	1.63 (1.13; 2.36)	1.63 (1.13; 2.36)
**Current living arrangement**			
Lives alone (reference)	1.00	1.00	1.00
Lives with family or friends	1.09 (0.79; 1.51)	1.02 (0.72; 1.46)	1.02 (0.72; 1.46)
**Marital status**			
Married/living together (reference)	1.00	1.00	1.00
Widowed/divorced/separated	1.49 (1.07; 2.08)	1.36 (0.94; 1.97)	1.36 (0.94; 1.97)
Single	0.92 (0.69; 1.22)	0.90 (0.66; 1.23)	0.90 (0.66; 1.22)
**Use of alcoholic beverages**			
No (Ref)		1.00	1.00
Yes		1.41 (1.03; 1.94)	**1.41 (1.03; 1.93)**^**c**^
**Substance use**			
No (reference)		1.00	1.00
Yes		1.12 (0.81; 1.55)	1.12 (0.80; 1.55)
**Use of tobacco products**			
No (reference)		1.00	1.00
Yes		1.37 (0.97; 1.94)	1.37 (0.97; 1.93)
**Blood pressure**			
Normal (reference)		1.00	1.00
High		0.95 (0.72; 1.25)	0.95 (0.72; 1.25)
**Body mass index**		1.01 (0.99; 1.04)	1.01 (0.99; 1.04)
**Negative life events**			
No (reference)		1.00	1.00
Yes		2.33 (1.73; 3.14)	**2.32 (1.72; 3.13)**^**d**^
**Chronic conditions**			
Arthritis		0.91 (0.58; 1.43)	0.91 (0.58; 1.43)
Chronic back/neck pain		1.53 (1.15; 2.04)	**1.53 (1.15; 2.04)**^**e**^
Frequent headaches		1.63 (1.25; 2.12)	**1.63 (1.25; 2.12)**^**f**^
Cancer		0.53 (0.16; 1.70)	0.53 (0.16; 1.70)
**Kessler psychological distress score**		1.17 (1.14; 1.19)	**1.17 (1.14; 1.19)**^**g**^
Past year Lifetime		0.49 (0.30; 0.79) 2.92 (2.11; 4.04)	**0.49 (0.30; 0.79)**^**h**^ **2.92 (2.11; 4.03)**^**i**^
**Country Geographical location**			
Southern Africa (reference)			1.00
Eastern Africa			0.63 (0.05; 7.13)
**ICC**	0.39 (0.12; 0.75)	0.24 (0.05; 0.65)	0.24 (0.05; 0.64)

*CI*confidence intervals, *aOR* adjusted odds ratios, *ICC* intraclass correlation coefficient.

* Multilevel (random-intercept) logistic regression; effect sizes are aOR with 95% CI, and ICC (95% CI) reflects between-country variance. All hypothesis tests are two-sided Wald z-tests (asymptotic; no degrees of freedom apply); no adjustment was made for multiple comparisons.

Superscripts denote exact two-sided Model 3 *P* values: ^a^*P* < 0.001; ^b^*P* = 0.021; ^c^*P* = 0.032; ^d^*P* < 0.001; ^e^*P* = 0.004; ^f^*P* < 0.001; ^g^*P* < 0.001; ^h^*P* = 0.004; ^i^*P* < 0.001.

## Discussion

### Prevalence

An overall prevalence of 0.9% was observed for current MDD, with country-specific prevalences ranging from 0.1 to 2.1% were observed across all the study countries. This observed prevalence is notably lower than the estimated global current prevalence of 3.4% MDD^2^. The prevalence of lifetime MDD in this study was 5.1%, also lower than the estimated lifetime prevalence of 14.6% in high-income countries and 11.1% in low- and middle-income countries^25^. However, this prevalence is comparable to previously reported prevalence estimates of 4.5% in SSA^2^ and 5.4% Africa overall^4^. The lower prevalence for MDD in SSA may be attributed to a complex interplay of cultural, social, and methodological factors. In many African cultures, the manifestation of MDD may differ from how it is traditionally defined or recognized in Western contexts. Tools developed in Western contexts may lack sensitivity to the cultural expression of depression in African populations, leading to potential underdiagnosis or misclassification. This challenge is further compounded by Africa’s immense cultural and linguistic diversity.

Additionally, the presence of unique cultural resilience factors and coping mechanisms may play a role in mitigating the onset of depressive symptoms or influencing their recognition and reporting. There is a pressing need to better understand how these factors interact to influence MDD development in African contexts.

Also, as expected, lifetime prevalence of MDD is higher than current prevalence due to the broader reference period it captures—any episode of MDD occurring across a person’s life, as well as the episodic nature of the disorder.

For South Africa, the observed current and lifetime prevalence of MDD (2.1% and 8.4%, respectively) are less than the prevalence of 4.9% and 9.8%, which have been reported for point and lifetime MDD, respectively, in South Africa^26, 27^. For Uganda, the observed current and lifetime prevalence of MDD (0.7% and 7.9 respectively) are much lower than the pooled prevalence of 20.8% which was reported among general populations in Uganda by a systematic review and meta-analysis^9^. For Ethiopia, the observed current and lifetime prevalence of MDD (0.5% and 1.2% respectively) are much lower than the prevalence of 5.3% and 17.4% which have been reported for current and lifetime MDD, respectively^28^. For Kenya, the observed current and lifetime prevalence of MDD (0.1% and 0.3% respectively) are much lower than the prevalence of 4.4% which has been reported among general populations in Kenya^8^.

The variations of prevalence by study country are intriguing and could be due to several factors such as socioeconomic factors, cultural differences, stigma, and awareness. Higher prevalence for South Africa and Uganda might correlate with socioeconomic stressors such as poverty, unemployment, and social inequality that can contribute to higher rates of depression. For example, South Africa has been reported to have a significant income inequality and high levels of unemployment^29^. Ethiopia and Kenya, with lower rates, might have different socioeconomic dynamics, or other protective factors at play, such as stronger community and family support systems. Cultural norms can influence how individuals express emotions or depression. In some cultures, it’s common to express MDD through physical symptoms rather than psychological terms, which could lead to underdiagnosis of MDD. There is a need to understand potential ecological factors responsible for variations in the prevalence of MDD across various regions in SSA.

### Factors associated with major depressive disorder

A per-year increase in age was associated with lower odds for lifetime MDD. Directions of associations between age and depression have been inconsistent. A large literature review and meta-analysis has reported depression to be more prevalent among elderly populations^30^ while analysis of large national health data from the United States has reported MDD to be more prevalent among young adults aged 18–29 years^31^. There is no clear explanation for the association between increasing age and lower odds for MDD in our study participants. However, several factors such as socio-cultural differences, socioeconomic, demographic and access to mental health services could be responsible. For example, there may be better economic conditions and pension schemes for older adults, and there may be more awareness and access to mental health care for older adults as compared to young adults. Younger adults in SSA may face more socioeconomic hardships and unemployment. There is a need to understand how these factors may influence the risk for MDD across the lifespan in SSA in order to inform targeted mental health interventions.

Male sex at birth was associated with lower odds for MDD. This finding is in line with findings from a large systematic analysis of the global burden of MDD in 2014 between 1990 and 2019, which reported MDD burden to be higher among females as compared with males^2^. Additionally, a large systematic review and meta-analysis of the epidemiology of MDD among people living with HIV in SSA, also reported the prevalence of MDD to be much higher among women as compared to men^32^. Although the underlying biological mechanisms for sex disparity in MDD are not well known, sex hormones have been postulated to play a role. In addition to the role of genetics and environmental factors like stress, sex hormone status has been postulated to be a third factor which contributes to changes in the epigenome, whose effect may translate into changes in gene expression and brain structure and function^33^, thus resulting in increased vulnerability for MDD in women.

Alcohol use was associated with higher odds for MDD. This finding is in line with findings from a large systematic review and meta-analysis of cohort studies, which reported an association between alcohol use and MDD^34^. However, it was observed that this association became nonsignificant when confounders were controlled for, suggesting that alcohol use could be leading to MDD through confounders. For example, unemployed individuals are more likely to abuse drugs and be heavy alcohol users and to suffer from MDD^35^.

Witnessing or experiencing traumatic life events was associated with higher odds for MDD. This finding is in agreement with findings from previous studies which reported traumatic life events to have a strong relationship with depression^36^ including studies in Uganda that found an increased risk of MDD with increasing negative life events^11,37^. Previous studies have also found that recent traumatic life events play a key role in the onset of MDD^38^. However, due to a lack of data, the onset of MDD and the timing of the negative life events were not determined by this study, and so this could not be properly investigated.

Chronic neck or back pain was associated with higher odds for MDD. This finding is in line with previous studies, which have reported associations between chronic pain and depression and have proposed that this could be mediated through altered neuroplasticity^39^. This relationship has been found to be influenced by anomalies in neurological function that lead to chronic pain preceding depression and depressive symptoms^39^. Back pain in general has been associated with increased risk for MDD among middle-aged adults in six countries, including Ghana and South Africa^40^. Additionally, a study in southwestern Uganda also found an association between reported back pain and MDD among elderly persons living with HIV/AIDS^41^.

Frequent headaches were associated with higher odds for MDD. This finding is in agreement with findings from a systematic review and meta-analysis which reported migraines (frequent headaches) to be a risk factor for incident cases of MDD in both cross-sectional and cohort studies^42^. However, this relationship could be bidirectional as MDD has also been found to be causal to different forms of frequent headaches by a Mendelian randomization study^43^. Frequent headaches have been suggested to lead to or exacerbate MDD through neurotransmitter imbalances, particularly serotonin and norepinephrine, which affect both pain perception and mood^44^. Chronic pain from headaches can impact daily life and contribute to the development or worsening of MDD.

A per-unit increase in K10 score was associated with higher odds for both current and lifetime MDD. This is not surprising as the K10 score measures psychological distress and the K10 tool has items which assess for both depression and anxiety symptoms a person has experienced in the most recent 4-week period^45^. If validated, the K10 score could be used as a tool that can identify people who are at risk of developing MDD in SSA for early intervention to reduce the overall burden of MDD on individuals and healthcare systems.

Lifetime positive screening for psychosis was associated with higher odds for MDD. This could be due to a fact that some people who suffer from MDD normally report symptoms of psychosis such as delusions and hallucinations^46^. The form of MDD with psychotic features is termed psychotic depression according to the International Classification of Diseases 11^th^ revision^47^. Psychotic depression has been reported to be a risk factor for psychosis in patients whose index depression had psychotic features^48^ hence the need for effective preventative treatments. Psychotic depression is hard to treat and has been reported to be unresponsive to an antidepressant alone^46^. Elevated levels of cortisol have been suggested to be responsible for this form of depression^49,50^. Paradoxically, a past year positive screening for psychosis was associated with lower odds for MDD. There is no direct explanation for this observation. However, acute management and treatment could mitigate the symptoms of MDD. Indeed, atypical antipsychotics have been reported to have a direct preventive effect on the development of depressive symptoms during management of acute psychosis^51^.

The proportion of variance in the prevalence of MDD across the study countries was moderate (14%) for current MDD and moderately large (24%) for lifetime MDD. There is thus a need to understand the specific risk factors for MDD that operate not only at the individual level but also across different national and regional contexts in SSA. Also, given that a moderately large proportion of variance is contributed by country-level factors, intervention strategies targeting structural and policy-level determinants may be more feasible and effective in mitigating MDD than individual-level strategies, particularly in resource-constrained settings. Examples of such country-level strategies include integrating mental health services into primary healthcare systems, expanding the mental health workforce through task-shifting, implementing national mental health policies, and investing in community-based mental health infrastructure. The higher proportion in variance observed for lifetime MDD further suggests that persistent regional-level factors such as historical exposure to conflict, socioeconomic inequalities, cultural stigma around mental health, and disparities in health financing and service availability, that accumulate over the lifespan. Targeting these broader determinants through systemic interventions may yield more sustainable improvements in mental health outcomes than relying solely on individualized care models.

### Limitations

We could not elucidate the causation of MDD given the cross-sectional nature of the study. Also, in assessing life-time MDD, we relied on the respondents’ memory and recall bias which may potentially have resulted in a systematic bias against the recall of temporally distant events. Additionally, while the MINI is a widely recognized tool for assessing MDD and provides a standardized criterion based on the diagnostic and statistical manual of mental disorders—fifth edition (DSM-5), it may not capture all culturally nuanced manifestations of depression, and its reliance on participants’ responses poses a risk of misclassfications—it is not a substitute for a full clinical diagnosis by a mental health professional. Future studies should endeavor to validate assessment tools for MDD for their use in SSA. Also, the study population was recruited from individuals present at general medical hospitals, including hospital staff, caretakers accompanying patients, and individuals attending outpatient clinics. As such, the sample may not be representative of the general population in the respective countries. Individuals present in hospital settings may have greater access to healthcare services, higher health literacy, or different socioeconomic characteristics compared to community-based populations. Furthermore, people with MDD or those who avoid healthcare settings may have been underrepresented. Consequently, the prevalence estimates reported in this study should not be interpreted as population-level prevalence estimates, and the identified associations should be understood as reflecting patterns within hospital-recruited adult populations rather than nationally representative samples. Also, since the diagnostic tool used was developed in Western contexts, this could be less sensitive to the cultural expression of MDD in African populations hence leading to potential misdiagnosis. Future multi-country population-based studies are needed to better generalize findings across broader populations. Also, due to a lack of data, we did not examine the effect of socioeconomic status on the observed association, despite socioeconomic factors being widely recognized as important determinants of mental health, including MDD. Future studies should prioritize the collection of this data to better understand how social determinants interact with clinical and demographic factors in shaping MDD outcomes in Africa.

This study investigated several factors associated with MDD in participants present at general medical facilities from a majorly African ancestry population. The main findings from the phenotypic analysis were that among the study participants, being female, using alcohol, experiencing or witnessing negative life events, having higher levels of psychological distress and suffering from chronic conditions of either chronic back or neck pain were associated with higher odds for MDD while per year increase in age was associated with lower odds for depression among the study participants. These results suggest that psychosocial factors, as well as psychosomatic complaints and physical comorbidities, are important risk factors for MDD among the participants in this study.

## Methods

### Ethical considerations

This study complied with all relevant ethical regulations governing research involving human participants and was conducted with the principles of the World Medical Association (Declaration of Helsinki). Ethical and scientific clearance for the NeuroGAP-Psychosis study was obtained from each of the study sites as shown in the following sentences; Uganda: The Makerere University School of Medicine Research and Ethics Committee (SOMREC #REC REF 2016-057) and the Uganda National Council for Science and Technology (UNCST #HS14ES); Kenya: Moi University College of Health Sciences/Moi Teaching and Referral Hospital Institutional Research and Ethics Committee (IREC) (#IREC/2016/145, approval number: IREC 1727), Kenya National Council of Science and Technology (#NACOSTI/P/17/56302/19576), KEMRI Centre Scientific Committee (CSC#KEMRI/CGMRC/CSC/070/2016), KEMRI Scientific and Ethics Review Unit (SERU# KEMRI/SERU/CGMR-C/070/3575); Ethiopia: Addis Ababa University College of Health Sciences (#014/17/Psy) and the Ministry of Science and Technology National Research Ethics Review Committee (#3.10/14/2018); South Africa: The University of Cape Town Human Research Ethics Committee (#466/2016); and USA: The Harvard T.H. Chan School of Public Health (#IRB17-0822). Participants provided written informed consent for their genetic and health information to be used in future research. Participants’ priorities and experiences were taken into consideration during the design of the NeuroGAP-Psychosis study. All participants were compensated in monetary terms for their transport and time in accordance with local study procedures.

### Study design

**Fig. 1:**
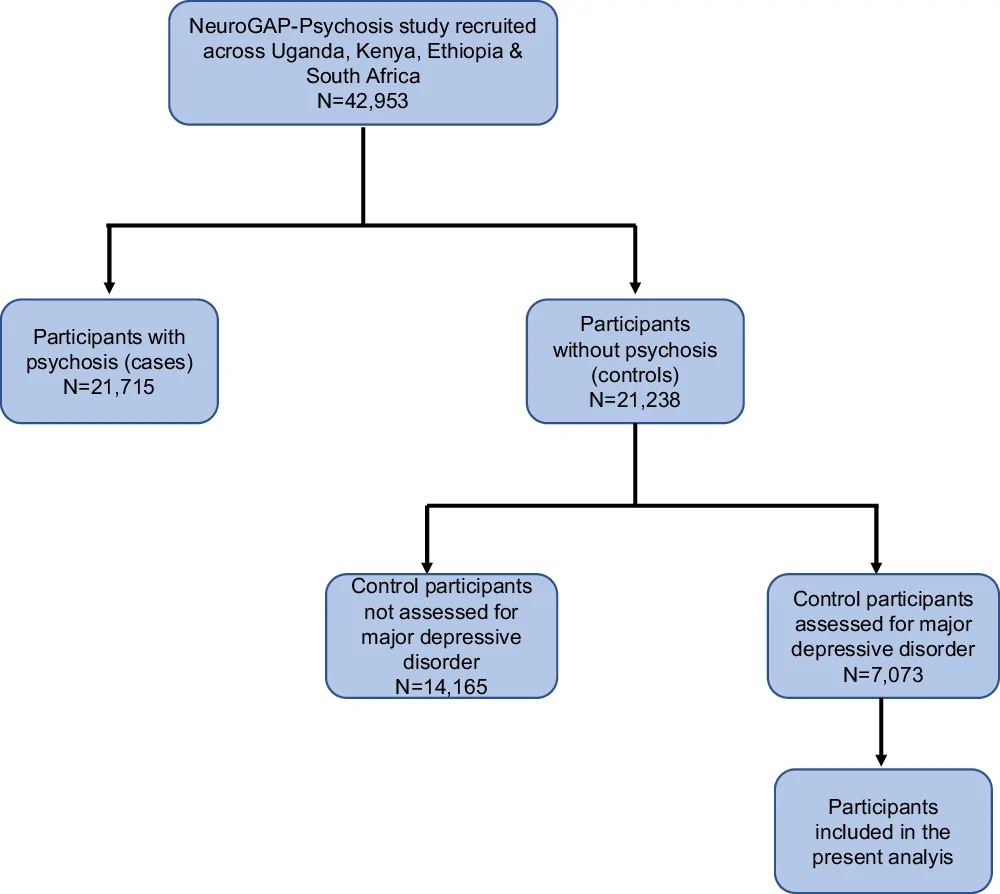
Flow diagram of participant selection from the NeuroGAP-Psychosis study cohort. Flow diagram showing the derivation of the analytic sample used in this study. Control participants assessed for major depressive disorder were included in the present analysis.

This study was undertaken using data collected by the NeuroGAP-Psychosis study. Details about the NeuroGAP-Psychosis study, the data variables collected, and the tools used have been reported by Stevenson and colleagues^52^. In brief, the NeuroGAP-Psychosis study recruited a total of 42,953 participants (21,715 cases of psychosis, 21,238 psychosis-free controls) from four African countries of Uganda, Kenya, Ethiopia, and South Africa over a 5-year period (2018 – 2023). The aim of the NeuroGAP-Psychosis study is to investigate the genetic risk for psychosis. Each study participant provided a saliva sample from which DNA was extracted and shipped to the Broad Institute in the United States, where it was sequenced. The exclusion criteria for NeuroGAP-Psychosis study’s control participants were: 1) having a psychotic disorder diagnosis or history of psychotic symptoms, 2) currently taking psychiatric medication for psychosis, 3) acute levels of alcohol use or substance abuse (i.e., use of psychoactive substances), as demonstrated by being a current inpatient or under acute medical care for these disorders, not fluent in one of the languages the consent form was translated into and not being able to consent to the study as defined by the University of California, San Diego Brief Assessment of Capacity to Consent questionnaire (UBACC)^53^. Although individuals with a diagnosed psychotic disorder or those receiving treatment for psychosis were excluded from the NeuroGAP-Psychosis control group, screening instruments for psychotic experiences and substance use were still administered to all participants as part of the broader assessment battery. These measures capture subthreshold symptoms or lifetime experiences rather than clinical diagnoses. Similarly, while individuals with acute substance misuse requiring clinical care were excluded, self-reported alcohol consumption and substance use behaviors were still assessed among participants. Consequently, the presence of positive psychosis screening responses or reports of alcohol and substance use in the present analysis reflects subclinical experiences or behavioral exposures within the control population rather than diagnosed psychotic disorders or acute substance misuse. All NeuroGAP-Psychosis study’s control participants were systematically assessed for current and lifetime MDD using the MINI^24^ with no additional selection process and are included in this analysis. Because assessment for MDD was integrated into the NeuroGAP-Psychosis study protocol partway through the study, only 7,073 out of the total 21,238 control participants were assessed for MDD. The derivation of this study’s analytic sample from the parent NeuroGAP-Psychosis cohort is shown in Fig. 1. The NeuroGAP-Psychosis study’s control participants were recruited from adults present at University-affiliated general medical hospitals that serve catchment areas similar to those of the psychiatric facilities where the psychosis cases for the NeuroGAP-Psychosis study were recruited. NeuroGAP-Psychosis study control participants were not patients seeking care for their own medical conditions. Rather, they comprised individuals present in the hospital environment for other reasons, including 1) caretakers who had accompanied someone else to an appointment, 2) students, interns, or staff working at the hospital/clinic, and 3) individuals present at the facility for routine or administrative purposes such as prescription refills. As such, the study population represents non-patient adults recruited from hospital environments rather than a population-based sample of adults in the respective countries. Individuals present in hospital settings may differ from the general population in several ways, including greater familiarity with healthcare systems, higher health awareness, or different socioeconomic and educational profiles. Consequently, the findings of this study should be interpreted as reflecting risk factors for major depressive disorder among adults recruited from hospital environments rather than estimates that are directly generalizable to national populations. This study was reported in accordance with the Strengthening the Reporting of Observational Studies in Epidemiology (STROBE) guidelines for cross-sectional studies.

### Clinical investigations

A questionnaire (module A of the MINI) was administered by trained research assistants to the study participants at each of the study sites. Assessment of MDD based on the presence of at least one of the symptoms of either a depressed mood or loss of interest in pleasurable activities and any other four symptoms, which may include significant weight change or appetite disturbance, sleep disturbances, psychomotor agitation or retardation, fatigue, feelings of worthlessness or excessive guilt, diminished ability to think or concentrate and recurrent thoughts of death or suicidal ideation. Additionally, these symptoms had to cause significant distress or impairment in social, occupational, or other important areas of functioning and were not a result of physiological effects of a substance or another medical condition. For current MDD, the presence of symptoms in the last two weeks was assessed, while for lifetime MDD, the presence of these symptoms in a participant’s lifetime was assessed. The MINI was selected due to its brevity, diagnostic alignment with the DSM-5 diagnostic criteria, and prior use in SSA. While the MINI was translated into local languages at the different sites and adapted as per the WHO guidelines, we recognize that such tools, originally developed in Western settings, may not fully capture local idioms of distress or the unique ways people in different cultures express symptoms. We, however, found the MINI to have a good internal reliability across the study countries (Cronbach’s alpha = 0.8). Nevertheless, the MINI’s cross-country use enabled diagnostic harmonization and comparability across the study countries. Participants who screened positive on the MINI were referred to a mental health specialist for further assessment and management. The five-item version of the psychosis screening questionnaire (PSQ)^54^ was used to screen for psychosis symptoms. The Kessler Psychological Distress Scale (K10)^45^ was used to assess for psychological distress. This is a ten-item questionnaire based on questions about symptoms of anxiety and depression. The Life Events Checklist for DSM-5 (LEC-5)^55^ was used to assess for negative life events. This checklist assesses exposure to sixteen events known to potentially result in post-traumatic stress disorder or distress. The World Health Organization Alcohol, Smoking and Substance Involvement Screening Test (ASSIST, version3)^56^ was used to assess for lifetime use of alcohol, tobacco and other substances. The Composite International Diagnostic Interview (CIDI) screener^57^ was used to assess for chronic conditions such as arthritis or rheumatism, chronic back or neck pain, frequent or severe headaches, cancer, among others. All data collections were captured in REDCap software.

### Data analysis

All statistical analyses were conducted using STATA version 18.0 (StataCorp LLC, College Station, TX, USA). Descriptive statistics were used to summarize socio-demographic characteristics, including sex, age, marital status, living arrangement, education level, study country, and body mass index, as well as clinical characteristics, including tobacco use, alcohol use, substance use, and blood pressure. Categorical variables were summarized using frequencies and percentages, while continuous variables were reported as medians with interquartile ranges (IQR). The prevalence of both current and lifetime MDD was estimated for each study country, along with corresponding 95% confidence intervals.

To examine factors associated with MDD, we employed a multilevel logistic regression model to account for the hierarchical structure of the data, with individuals nested within four countries, and estimated intraclass correlation coefficients (ICCs). The ICCs reflect the proportion of variance attributable to between-country differences. Factors such as age, sex, level of education, alcohol use, and comorbid physical health conditions (e.g., high blood pressure), which have previously been associated with MDD, particularly in SSA, were selected for analysis. Conceptually, inclusion was guided by the biopsychosocial model of depression, which integrates biological, psychological, and social determinants of depression.

Model selection was guided by the likelihood ratio test (LRT) to ensure that only variables that significantly improved model fit were retained. The modeling process followed a sequential approach. First, model 1 included key socio-demographic variables (age, sex, education level, living arrangement, and marital status), selected a priori based on theoretical and empirical evidence. Model 2 extended Model 1 by incorporating individual-level behavioral and clinical characteristics, including alcohol use, substance use, history of traumatic life events, and current physical illness. Variables were retained only if their inclusion significantly improved model fit based on LRT. Finally, model 3 introduced country-level characteristics, such as geographical location, to assess their influence while controlling for individual-level factors.

The intraclass correlation coefficient (ICC) was calculated for each model to quantify the proportion of total variance attributable to between-country differences. A variable was considered meaningfully associated with MDD only if it remained statistically significant in the final model and contributed to the model fit, as determined by LRT. To assess the robustness of the findings, a sensitivity analysis was performed by systematically excluding individual countries and re-estimating the models. A two-sided *p*-value < 0.05 was considered statistically significant.

We assessed key statistical assumptions to ensure model validity. Multicollinearity was evaluated using variance inflation factors, and all values were within acceptable limits ( < 10), indicating no strong correlations among predictor variables. For continuous variables such as age, body mass index, and K10 scores, we assessed the linearity of the logistic regression model using visual plots and confirmed that the assumption was reasonably met. Intra-class correlation coefficients (ICCs) were calculated to account for clustering at the country level, and the normality of random effects was evaluated using quantile-quantile (Q-Q) plots. The final model specification was informed by likelihood ratio tests for variable selection.

### Reporting summary

Further information on research design is available in the Nature Portfolio Reporting Summary linked to this article.

## Supplementary information

Reporting Summary

Transparent Peer Review file

## Data Availability

The NeuroGAP-Psychosis cohort data underlying this study have been deposited in the National Institute of Mental Health Data Archive (NDA) under Study Accessions #3805 (NDA-GRU), #4538 (Disease-Specific (Mental Health)), and #4539 (NDA-HMB-MDS). Access to these data is currently under embargo until July 2027 in accordance with the NeuroGAP-Psychosis data-sharing policy and agreements with participating investigators, institutions, and the National Institute of Mental Health. Following the embargo, qualified researchers will be able to request access through the NDA in accordance with its data access procedures. During the embargo period, requests for access should be directed to Mr. Rocky Stroud at rstroud@hsph.harvard.edu or the NeuroGAP administrative team at neurogapadmin@hsph.harvard.edu.
