# Supplementary material for: Risk factors for major depressive disorder among adults recruited from hospital settings in Uganda, Kenya, Ethiopia and South Africa
Source: Nat Commun. 2026 Aug 27;17:10270. doi: 10.1038/s41467-026-77189-6 (PMC13616959; doi:10.1038/s41467-026-77189-6)
Supplement: Supplementary file 1 [file 41467_2026_77189_MOESM1_ESM.pdf]

nature portfolio

Corresponding author(s): Allan Kalungi

Last updated by author(s): 2026 07 10

Reporting Summary

Nature Portfolio wishes to improve the reproducibility of the work that we publish. This form provides structure for consistency and transparency in reporting. For further information on Nature Portfolio policies, see our Editorial Policies and the Editorial Policy Checklist.

Statistics

For all statistical analyses, confirm that the following items are present in the figure legend, table legend, main text, or Methods section.

n/a	Confirmed	
x		The exact sample size (*n*) for each experimental group/condition, given as a discrete number and unit of measurement
x		A statement on whether measurements were taken from distinct samples or whether the same sample was measured repeatedly
	x	The statistical test(s) used AND whether they are one- or two-sided<br>*Only common tests should be described solely by name; describe more complex techniques in the Methods section.*
	x	A description of all covariates tested
x		A description of any assumptions or corrections, such as tests of normality and adjustment for multiple comparisons
	x	A full description of the statistical parameters including central tendency (e.g. means) or other basic estimates (e.g. regression coefficient) AND variation (e.g. standard deviation) or associated estimates of uncertainty (e.g. confidence intervals)
	x	For null hypothesis testing, the test statistic (e.g. *F*, *t*, *r*) with confidence intervals, effect sizes, degrees of freedom and *P* value noted<br>*Give P values as exact values whenever suitable.*
x		For Bayesian analysis, information on the choice of priors and Markov chain Monte Carlo settings
	x	For hierarchical and complex designs, identification of the appropriate level for tests and full reporting of outcomes
x		Estimates of effect sizes (e.g. Cohen's *d*, Pearson's *r*), indicating how they were calculated

Our web collection on statistics for biologists contains articles on many of the points above.

Software and code

Policy information about availability of computer code

Data collection	REDcap electronic data capture tools were used for electronic data collection.
Data analysis	Statistical analyses were performed using Stata version 18.0 (StataCorp LLC, College Station, TX, USA).

For manuscripts utilizing custom algorithms or software that are central to the research but not yet described in published literature, software must be made available to editors and reviewers. We strongly encourage code deposition in a community repository (e.g. GitHub). See the Nature Portfolio guidelines for submitting code & software for further information.

Data

Policy information about availability of data

All manuscripts must include a data availability statement. This statement should provide the following information, where applicable:

- Accession codes, unique identifiers, or web links for publicly available datasets
- A description of any restrictions on data availability
- For clinical datasets or third party data, please ensure that the statement adheres to our policy

The NeuroGAP-Psychosis cohort data underlying this study have been deposited in the National Institute of Mental Health Data Archive (NDA) under Study Accessions #3805 (NDA-GRU), #4538 (Disease-Specific (Mental Health)), and #4539 (NDA-HMB-MDS). Access to these data is currently under embargo until July 2027 in accordance with the NeuroGAP-Psychosis data-sharing policy and agreements with participating investigators, institutions and the National Institute of Mental Health. Following the embargo, qualified researchers will be able to request access through the NDA in accordance with its data access procedures. During the embargo period, requests for access should be directed to Mr Rocky Stroud at rstroud@hsph.harvard.edu or the NeuroGAP administrative team at

neurogapadmin@hsph.harvard.edu.

Research involving human participants, their data, or biological material

Policy information about studies with human participants or human data. See also policy information about sex, gender (identity/presentation), and sexual orientation and race, ethnicity and racism.

Reporting on sex and gender	Sex was self-reported and recorded as sex at birth. The study included 2,259 females and 4,814 males. Sex was considered during study design and included as a covariate in all multivariable analyses.
Reporting on race, ethnicity, or other socially relevant groupings	We only reported on country and geographical region where participants resided. Participants were categorized according to country of recruitment and geographical region. The study included participants from Uganda, Kenya, Ethiopia and South Africa. Uganda, Kenya and Ethiopia were classified as Eastern Africa, while South Africa represented Southern Africa.
Population characteristics	See the section for behavioural and social sciences research design, below.
Recruitment	The study population was recruited from individuals present at general medical hospitals, including hospital staff, caretakers accompanying patients and individuals attending outpatient clinics. As such, the sample may not be representative of the general population in the respective countries. Individuals present in hospital settings may have greater access to healthcare services, higher health literacy, or different socioeconomic characteristics compared to community-based populations. Furthermore, people with major depressive disorder or those who avoid healthcare settings may have been underrepresented. Consequently, the prevalence estimates reported in this study should not be interpreted as population-level prevalence estimates, and the identified associations should be understood as reflecting patterns within hospital-recruited adult populations rather than nationally representative samples.
Ethics oversight	The NeuroGAP-Psychosis study was conducted in compliance with the Code of Ethics of the World Medical Association (Declaration of Helsinki). Ethical and scientific clearance for the NeuroGAP-Psychosis study was obtained from each of the study sites as shown in the following sentences; Uganda: The Makerere University School of Medicine Research and Ethics Committee (SOMREC #REC REF 2016-057) and the Uganda National Council for Science and Technology (UNCST #HS14ES); Kenya: Moi University College of Health Sciences/Moi Teaching and Referral Hospital Institutional Research and Ethics Committee (IREC) (#IREC/2016/145, approval number: IREC 1727), Kenya National Council of Science and Technology (#NACOSTI/P/17/56302/19576), KEMRI Centre Scientific Committee (CSC#KEMRI/CGMRC/CSC/070/2016), KEMRI Scientific and Ethics Review Unit (SERU# KEMRI/SERU/CGMR-C/070/3575); Ethiopia: Addis Ababa University College of Health Sciences (#014/17/Psy) and the Ministry of Science and Technology National Research Ethics Review Committee (#3.10/14/2018); South Africa: The University of Cape Town Human Research Ethics Committee (#466/2016); and USA: The Harvard T.H. Chan School of Public Health (#IRB17-0822). Participants provided written informed consent for their genetic and health information to be used in future research. Participants' priorities and experiences were taken into consideration during the design of the NeuroGAP-Psychosis study. All participants were compensated in monetary terms for their transport and time in accordance with local study procedures.

Note that full information on the approval of the study protocol must also be provided in the manuscript.

Field-specific reporting

Please select the one below that is the best fit for your research. If you are not sure, read the appropriate sections before making your selection.

☐ Life sciences ☒ Behavioural & social sciences ☐ Ecological, evolutionary & environmental sciences

For a reference copy of the document with all sections, see nature.com/documents/nr-reporting-summary-flat.pdf

Behavioural & social sciences study design

All studies must disclose on these points even when the disclosure is negative.

Study description	This is a quantitative, multi-country cross-sectional study using harmonized psychiatric assessments to investigate the prevalence and risk factors for current and lifetime major depressive disorder among adults recruited from hospital settings in four African countries.
Research sample	The study population was recruited from adults present at general medical hospitals, including hospital staff, caretakers accompanying patients and individuals attending outpatient clinics, as part of the NeuroGAP-Psychosis study. This sample was selected because all psychosis-free control participants who completed the MINI assessment for major depressive disorder were included in this secondary analysis, enabling standardized cross-country comparisons using harmonized data collected under a common protocol. As such, the sample may not be representative of the general population in the respective countries. Individuals present in hospital settings may have greater access to healthcare services, higher health literacy or different socioeconomic characteristics compared with community-based populations. Furthermore, people with major depressive disorder or those who avoid healthcare settings may have been underrepresented. Consequently, the prevalence estimates reported in this study should not be interpreted as population-level prevalence estimates, and the identified associations should be understood as reflecting patterns within hospital-recruited adult populations rather than nationally representative samples.
Sampling strategy	Adult participants recruited into the NeuroGAP-Psychosis control cohort at participating hospitals were systematically assessed for current and lifetime major depressive disorder using the Mini International Neuropsychiatric Interview (MINI version 7.0.2). All eligible participants with complete depression assessment data were included in this analysis.

Data collection	Data were collected by trained research assistants using interviewer-administered structured questionnaires programmed into REDCap electronic data capture tools. Standardized instruments included the Mini International Neuropsychiatric Interview (MINI version 7.0.2), Kessler Psychological Distress Scale (K10), Psychosis Screening Questionnaire (PSQ), Life Events Checklist for DSM-5 (LEC-5), WHO ASSIST version 3, the Composite International Diagnostic Interview (CIDI) screener, and structured demographic and clinical questionnaires. Interviews were conducted in private settings within participating hospitals.
Timing	Data collection was conducted between 2017 and 2023.
Data exclusions	Participants with complete data for the variables included in each analysis were included. No additional exclusion criteria were applied beyond those described in the Methods.
Non-participation	Information on participant non-participation or refusal rates was not systematically recorded across study sites.
Randomization	Not applicable. This was an observational cross-sectional study with no allocation of participants to experimental groups.

Reporting for specific materials, systems and methods

We require information from authors about some types of materials, experimental systems and methods used in many studies. Here, indicate whether each material, system or method listed is relevant to your study. If you are not sure if a list item applies to your research, read the appropriate section before selecting a response.

Materials & experimental systems

n/a	Involved in the study	
☒	☐	Antibodies
☒	☐	Eukaryotic cell lines
☒	☐	Palaeontology and archaeology
☒	☐	Animals and other organisms
☐	☒	Clinical data
☒	☐	Dual use research of concern
☒	☐	Plants

Methods

n/a	Involved in the study	
☒	☐	ChIP-seq
☒	☐	Flow cytometry
☒	☐	MRI-based neuroimaging

Clinical data

Policy information about clinical studies

All manuscripts should comply with the ICMJE guidelines for publication of clinical research and a completed CONSORT checklist must be included with all submissions.

Clinical trial registration	Not applicable
Study protocol	Not applicable
Data collection	Not applicable
Outcomes	Not applicable

Plants

Seed stocks	Not applicable
Novel plant genotypes	Not applicable
Authentication	Not applicable
