# Supplementary material for: Risk factors for major depressive disorder among adults recruited from hospital settings in Uganda, Kenya, Ethiopia and South Africa
Source: Nat Commun. 2026 Aug 27;17:10270. doi: 10.1038/s41467-026-77189-6 (PMC13616959; doi:10.1038/s41467-026-77189-6)
Supplement: Supplementary file 2 [file 41467_2026_77189_MOESM2_ESM.pdf]

nature portfolio

Peer Review File

Risk factors for major depressive disorder among adults recruited from hospital settings in Uganda, Kenya, Ethiopia and South Africa

Corresponding Author: Dr Allan Kalungi

This file contains all reviewer reports in order by version, followed by all author rebuttals in order by version.

Version 0:

Reviewer comments:

Reviewer #1

(Remarks to the Author)
The manuscript focuses on the prevalence and risk factors of major depressive disorder among adults recruited in hospital settings in Ethiopia, Kenya, Uganda and South Africa. The manuscript addresses an important topic because the current knowledge on the nature and risk factors of depression in Africa is limited. However, I have a number of questions and suggestions.

General: If not required by the journal, I would place the methods section after the introduction and before the results section because this helps to understand many aspects mentioned in the results and discussion sections better.

Introduction
„Despite accumulating data on MDD prevalence across various regions in SSA, a comprehensive understanding of the disorder's epidemiological landscape remains elusive. Current research shows considerable variability in MDD prevalence. This suggests that existing studies may not fully capture the nuances of how depression manifests across SSA's varied cultural, economic, and demographic contexts. This may also be attributable to the different methods employed I in assessing for MDD and the different populations being sampled. Additionally, while links between MDD and lifestyle diseases such as diabetes and hypertension, as well as infectious diseases like HIV, Ebola, and COVID-19 have been noted, there is a dearth of studies exploring these relationships. The inconsistent use of diagnostic criteria and potential underdiagnosis due to cultural perceptions of mental health highlight the need for more localized and culturally adapted research methodologies."

In light of these important considerations, a weakness of the present study is that it did not use a culturally adapted measure of MDD that considers cultural expressions of symptoms. Hence, the study does not address this limitation which is emphasized in the introduction. In fact, the use of a diagnostic tool and criteria developed and validated in Western contexts is also presented as explanation for the relatively low prevalence rates of MDD observed in this study. I think this indicates a tautology related to the conceptual methodological approach of this study.

In a related vein, the unique contributions of this study should be emphasized more, pointing out limitations of specific existing epidemiological studies on MDD in Africa.

Please elaborate on the empirical and conceptual rationale for selecting the risk factors examined in this study.

Results
„In the multilevel models, male sex at birth was marginally significantly associated with current MDD (aOR = 0.47 [95% CI: 0.22; 1.02])."
Specify that this association was negative (lower odds).

Discussion
"This is need to understand how this factors interact to influence the development of MDD in Africa."
Please check the grammar in this sentence.

Paragraph starting on page 12, line 12: There are many repetitive elements in this section, .g., "..are much lower..". Consider revising.
Page 13: There is a typo in the sentence: "However, several factors such as ocio-cultural differences.."

"For Kenya, the observed current and lifetime prevalence of MDD (0.5% and 1.2% respectively) are much lower than the prevalence of 4.4% which has been reported among general populations in Kenya."
In table 2, the prevalence rates in Kenya are 0.1 and 0.3. Please clarify.

Socio-economic factors were frequently highlighted as potentially important in explaining the results, e.g., in relation to the associations between MDD and risk factors such as age and alcohol use. This raises the question why socioeconomic factors like income or employment were not considered in the analyses in the first place, especially because previous research also shows the importance of these factors with regard to depression.

"However, onset of MDD and the timing of the negative life events were not determined by the parent study and so this could not be properly investigated in this study."
I think there is a typo, it should be "present"

"The K10 score could be used as tool which can identify people who are at risk of developing MDD in SSA for early intervention to reduce the overall burden of MDD on individuals and healthcare systems."
It think this recommendation would require references to studies that validated the K10 in the study contexts.

"This could could be due to a fact that some people who suffer from MDD normally report symptoms of psychosis such as delusions and hallucinations."
"Could" is repeated twice.

"Also, given that moderately large proportion of variance is contributed by country level factors, country level intervention strategies could be effective in mitigating MDD rather than individual level strategies – which would likely be less feasible. In addition, the higher proportion in variance for lifetime MDD potentially suggests that persistent regional-level factors are influential and perhaps reflect long-standing regional-level characteristics, cultural factors or structural factors that affect MDD over the lifespan."
Could you provide examples for such country and regional level factors, perhaps based on previous research? What are examples for country level interventions? Why would they be more feasible than interventions targeting individual-level factors?

Limitations: Use of a self-report questionnaire rather than a clinical interview should also be considered a limitation. Furthermore, it should be pointed out that this assessment serves only as a screening for MDD, but does not allow for a diagnosis

Methods

How were the individuals who were assessed for MDD selected from the pool of control participants?

Clinical investigations: Please provide information on the reliability and validity of the tools in the study countries.

Ethical considerations
Please describe referral procedures for participants who screened positive for MDD.

Data analyses
I was wondering about the appropriateness of running a multilevel model with only four level 2 units (countries). In my opinion including country as fixed effect (dummy variable) would be more appropriate with such a small number of level 2 units.

I would also expect a description of the testing of statistical assumptions of regression analysis.

Reviewer #2

(Remarks to the Author)
The authors present a clear description of the prevalence and factors associated with major depressive disorder in four countries of Africa, a region in which limited epidemiological data has previously indicated alarming rates of depression and in which treatment availability is low. While this is an important area of research conducted in a large and diverse population, I have two significant concerns related to the methodology that greatly diminish the impact of this work.

For one, there is not a clear description of the measures used. Were they adapted at all? What languages were administered in each location? What is the evidence for validity in these populations and what was the reliability in the current sample? While only touched on in the discussion, it seems linguistic understanding and cultural relevance may have been a major issue. Such huge MDD prevalence differences across countries suggest that the MINI may not have worked the same across contexts.

Additionally, this sample is not representative of the general population of these countries. Hospital staff are more educated and likely have greater awareness of depression, and people seeking care also likely have a physical health issue, which here and in other studies is associated with depression. Were the caretakers those of people with psychosis? That too would likely bias prevalence.

Considering the much lower prevalence of MDD identified in this study compared to others conducted in the region, I am concerned these issues have significantly impacted the results and made difficult their interpretation.

A few more minor comments below:

The abstract should note the much lower prevalence identified here than in prior research and a potential reasoning for why.

The association of sex and current MDD is inversed. Males are significantly LESS likely to have current MDD. The way it is written now (line 140) suggests they have higher odds.

The first mention of the Eastern and Southern African categories (line 153) should indicate which country was included in each category (ex. Uganda could be considered both).
Typo: Line 108, 167, 207, 246, 387

Open Access This Peer Review File is licensed under a Creative Commons Attribution 4.0 International License, which permits use, sharing, adaptation, distribution and reproduction in any medium or format, as long as you give appropriate credit to the original author(s) and the source, provide a link to the Creative Commons license, and indicate if changes were made.
In cases where reviewers are anonymous, credit should be given to 'Anonymous Referee' and the source.
The images or other third party material in this Peer Review File are included in the article's Creative Commons license, unless indicated otherwise in a credit line to the material. If material is not included in the article's Creative Commons license and your intended use is not permitted by statutory regulation or exceeds the permitted use, you will need to obtain permission directly from the copyright holder.
To view a copy of this license, visit https://creativecommons.org/licenses/by/4.0/

2nd December, 2025

Dear Editor,

Thank you so much for giving us the opportunity to revise and resubmit the manuscript. We have revised the manuscript to address the reviewers' comment and below is a detail the revisions we have made.

We hope that you will find our revisions and response satisfactory and you consider the manuscript to be published in this very prestigious journal.

Reviewer #1 (Remarks to the Author):
The manuscript focuses on the prevalence and risk factors of major depressive disorder among adults recruited in hospital settings in Ethiopia, Kenya, Uganda and South Africa. The manuscript addresses an important topic because the current knowledge on the nature and risk factors of depression in Africa is limited. However, I have a number of questions and suggestions.

General: If not required by the journal, I would place the methods section after the introduction and before the results section because this helps to understand many aspects mentioned in the results and discussion sections better.

Response:

Thank you for the suggestion. We agree that placing the Methods section before the Results can often aid reader comprehension. However, as per Nature Communications submission guidelines, the Methods section is placed after the Discussion. To maintain consistency with the journal's required structure, we have followed their formatting guidelines.

Introduction
„Despite accumulating data on MDD prevalence across various regions in SSA, a comprehensive understanding of the disorder's epidemiological landscape remains elusive. Current research shows considerable variability in MDD prevalence. This suggests that existing studies may not fully capture the nuances of how depression manifests across SSA's varied cultural, economic, and demographic contexts. This may also be attributable to the different methods employed I in assessing for MDD and the different populations being sampled. Additionally, while links between MDD and lifestyle diseases such as diabetes and hypertension, as well as infectious diseases like HIV, Ebola, and COVID-19 have been noted, there is a dearth of studies exploring these relationships. The inconsistent use of diagnostic criteria and potential underdiagnosis due to cultural perceptions of mental health highlight the need for more localized and culturally adapted research methodologies."

In light of these important considerations, a weakness of the present study is that it did not use a culturally adapted measure of MDD that considers cultural expressions of symptoms. Hence, the study does not address this limitation which is emphasized in the introduction. In fact, the use of a diagnostic tool and criteria developed and validated in Western contexts is also presented as explanation for the relatively low prevalence rates of MDD observed in this

study. I think this indicates a tautology related to the conceptual methodological approach of this study.

__Response:__

We appreciate this insightful comment. We agree that while the introduction emphasizes the importance of culturally adapted assessments, this was not fully addressed in the original justification of our measurement approach. However, we selected the MINI because of its brevity, diagnostic alignment with the DSM-5 diagnostic criteria and prior use in sub-Saharan Africa. We have also found the MINI to have a good internal reliability across the study countries (Cronbach's alpha = 0.8) in the present study. To clarify and address this potential tautology, we have revised the manuscript in the following ways:

1) *We have highlighted in the methods section why the MINI was used while acknowledging limitation of Western derived tools. We also now report the internal consistency of the tool in the revised manuscript (page 20, lines 444 – 453):*

"The MINI was selected due to its brevity, diagnostic alignment with the DSM-5 diagnostic criteria and prior use in SSA. While the MINI was translated into local languages at the different sites and adapted as per the WHO guidelines, we recognize that such tools, originally developed in Western settings may not fully capture local idioms of distress or the unique ways people in different cultures express symptoms. We however found the MINI to have a good internal reliability across the study countries (Cronbach's alpha = 0.8). Nevertheless, the MINI's cross-country use enabled diagnostic harmonization and comparability across the study countries. Participants who screened positive on the MINI were referred to a mental health specialist for further assessment and management."

2) *We also acknowledge in the limitations section that the MINI may not capture all the culturally nuanced manifestations of MDD (page 18, lines 371 – 376):*

"Additionally, while the MINI is a widely recognized tool for assessing MDD and provides a standardized criteria based on the diagnostic and statistical manual of mental disorders—fifth edition (DSM-5), it may not capture all culturally nuanced manifestations of depression and its reliance on participants' responses poses risk of misclassfications—it is not a substitute for full clinical diagnosis by a mental health professional." Future studies should endeavour to validate assessment tools for MDD for their use in SSA.

In a related vein, the unique contributions of this study should be emphasized more, pointing out limitations of specific existing epidemiological studies on MDD in Africa.

__Response:__

Thank you for this constructive suggestion. We agree that clearly articulating the unique contributions of our study—particularly in relation to limitations of previous research on MDD in Africa is important for contextualizing its value. In response, we have revised the Introduction to highlight key methodological and empirical gaps in existing studies, and to clarify how our approach addresses these (Last paragraph, pages 5, lines 125 – 134):

"While previous studies have contributed valuable insights into the burden of MDD in SSA, most have been limited by narrow geographical focus, non-standardized tools, and restricted population subgroups such as post-conflict or HIV-specific populations. Additionally, the inconsistent use of structured diagnostic interviews has hindered cross-study comparability and may have contributed to the wide variability in reported prevalence estimates. This study investigated the prevalence and correlates of MDD using a harmonized, multi-country dataset from the four African nations using a standardized Mini-International Neuropsychiatric Interview [20], enabling direct cross-national comparison. Moreover, by applying multilevel modeling, the study quantifies the extent of between-country variation in MDD and identifies both individual- and contextual-level risk factors."

Please elaborate on the empirical and conceptual rationale for selecting the risk factors examined in this study.

Response:

We appreciate this comment and we have now expanded the manuscript to clarify both the empirical and conceptual rationale for selecting the risk factors assessed in this study. Our selection was guided by existing literature on determinants of MDD, particularly in sub-Saharan African (SSA) settings, where factors such as age, sex, education level, alcohol use, and comorbid physical health conditions (e.g., hypertension) have been associated with MDD risk. Conceptually, we were guided by the biopsychosocial model of depression, which integrates biological, psychological, and social dimensions of vulnerability. We prioritized variables available across all cohorts that aligned with these domains and had evidence of relevance to MDD in SSA settings. These justifications have been incorporated into the revised manuscript under the data analysis section (page 22, lines 492 – 499):

"To examine factors associated with MDD, we employed a multilevel logistic regression model to account for the hierarchical structure of the data, with individuals nested within four different countries, while estimating the intraclass correlation coefficients (ICCs). The ICCs reflect the proportion of variance attributable to between-country differences. Factors such as age, sex, level of education, alcohol use and comorbid physical health conditions (e.g. high blood pressure) which have previously been associated with MDD particularly in SSA, were selected for analysis. Conceptionally, inclusion was guided by the biopsychosocial model of depression which integrates biological, psychological and social determinants of depression."

Results

„In the multilevel models, male sex at birth was marginally significantly associated with current MDD (aOR = 0.47 [95% CI: 0.22; 1.02])."

Specify that this association was negative (lower odds).

Response:

Thank you for this observation. We have revised this sentence (now lines 155 - 156, page 7):

"In the multilevel models, male sex at birth was marginally significantly associated with lower odds of current MDD (aOR = 0.47 [95% CI: 0.22; 1.02])."

Discussion
"This is need to understand how this factors interact to influence the development of MDD in Africa."
Please check the grammar in this sentence.

Response:

The grammar in this sentence has been corrected (line 229):

"There is a pressing need to understand how these factors ……."

Paragraph starting on page 12, line 12: There are many repetitive elements in this section, .g., "..are much lower..". Consider revising.

Response:

This paragraph has been revised and also broken down into three paragraphs for easy of readability (page 13, lines 208 – 235):

"An overall prevalence of 0.9% was observed for current MDD, with country-specific prevalences ranging from 0.1 to 2.1% were observed across all the study countries. This observed prevalence is notably lower than the estimated global current prevalence of 3.4% MDD.[3] The prevalence of lifetime MDD in this study was 5.1%, also lower than the estimated lifetime prevalence of 14.6% in high-income countries and 11.1% in low- and middle-income countries.[20] However, this prevalence is comparable to previously reported prevalence estimates of 4.5% in SSA [3] and 5.4% Africa overall.[5] The lower prevalence for MDD in SSA may be attributed to a complex interplay of cultural, social and methodological factors. In many African cultures, the manifestation of MDD may differ from how it is traditionally defined or recognized in Western contexts. Tools developed in Western contexts may lack sensitivity to the cultural expression of depression in African populations, leading to potential underdiagnosis or misclassification. This challenge is further compounded by Africa's immense cultural and linguistic diversity.

Additionally, the presence of unique cultural resilience factors and coping mechanisms may play role in mitigating the onset of depressive symptoms or influencing their recognition and reporting. There is a pressing need to better understand how these factors interact to influence the development of MDD in African contexts.

Also, as expected, lifetime prevalence of MDD is higher than current prevalence due to the broader reference period it captures—any episode of MDD occurring across a person's life, a well as the episodic nature of the disorder."

Page 13: There is a typo in the sentence: "However, several factors such as ocio-cultural differences.."

Response:

The typo in the sentence has been corrected (line 270):

"However, several factors such as socio-cultural differences …"

"For Kenya, the observed current and lifetime prevalence of MDD (0.5% and 1.2% respectively) are much lower than the prevalence of 4.4% which has been reported among general populations in Kenya."
In table 2, the prevalence rates in Kenya are 0.1 and 0.3. Please clarify.

Response:

Thank you for this observation. The rates we referenced (0.5% current and 1.2% lifetime MDD) were mistakenly attributed to Kenya, when in fact they are for Ethiopia. We have corrected this error (page 14, line 245):

"………. the observed current and lifetime prevalence of MDD (0.1% and 0.3% respectively) are ….."

Socio-economic factors were frequently highlighted as potentially important in explaining the results, e.g., in relation to the associations between MDD and risk factors such as age and alcohol use. This raises the question why socioeconomic factors like income or employment were not considered in the analyses in the first place, especially because previous research also shows the importance of these factors with regard to depression.

Response:

We acknowledge that socioeconomic factors are important in understanding the risk of MDD and their omission from the current analysis is a notable limitation. Unfortunately, these variables were not collected across the study sites within the NeuroGAP study which restricted our ability to include them in the analysis. We have highlighted this as a limitation to the study (pages 18, lines 387 – 392):

Also, due to lack of data, we did not examine the effect of socioeconomic status on the observed association, despite socioeconomic factros being widely recognized as important determinants of mental health , including MDD. Future studies should prioritize collection of this data to better understand how social determinants interact with clinical and demographic factors in shaping MDD outcomes in Africa.

"However, onset of MDD and the timing of the negative life events were not determined by the parent study and so this could not be properly investigated in this study."
I think there is a typo, it should be "present"

Response:

This was not a typo, we meant that data was not collected by the NeuroGAP study. This sentence has been revised to clarify this (page16, lines 301 – 303):

"However, due to lack of data, onset of MDD and the timing of the negative life events were not determined by this study and so this could not be properly investigated."

"The K10 score could be used as tool which can identify people who are at risk of developing MDD in SSA for early intervention to reduce the overall burden of MDD on individuals and healthcare systems."
It think this recommendation would require references to studies that validated the K10 in the study contexts.

Response:

Thank you so much for this comment, we have now put a conditional clause on this recommendation (page 16, line 328):

"If validated, the K10 score could be used as tool which can identify people who are at risk of developing MDD in SSA for early intervifention to reduce the overall burden of MDD on individuals and healthcare systems."

"This could could be due to a fact that some people who suffer from MDD normally report symptoms of psychosis such as delusions and hallucinations."
"Could" is repeated twice.

Response:

This has been corrected (page 16, lines 332 - 333).

"Also, given that moderately large proportion of variance is contributed by country level factors, country level intervention strategies could be effective in mitigating MDD rather than individual level strategies – which would likely be less feasible. In addition, the higher proportion in variance for lifetime MDD potentially suggests that persistent regional-level factors are influential and perhaps reflect long-standing regional-level characteristics, cultural factors or structural factors that affect MDD over the lifespan."
Could you provide examples for such country and regional level factors, perhaps based on previous research? What are examples for country level interventions? Why would they be more feasible than interventions targeting individual-level factors?

Response:

Thank you so much for this useful comment. We have revised the discussion to highlight examples of these country-level strategies and how they would be more feasible in mitigating MDD other than individual-level factors (page 17, last paragraph, lines 347 – 363):

"There is thus a need to understand the specific risk factors for MDD that operate not only at the individual level but also across different national and regional contexts in SSA. Also, given

that moderately large proportion of variance is contributed by country level factors, intervention strategies targeting structural and policy-level determinants may be more feasible and effective in mitigating MDD than individual level strategies, particularly in resource-constrained settings. Examples of such country-level strategies include integrating mental health services into primary healthcare systems, expanding the mental health workforce through task-shifting, implementing national mental health policies, and investing in community-based mental health infrastructure. The higher proportion in variance observed for lifetime MDD further suggests that persistent regional-level factors such as historical exposure to conflict, socioeconomic inequalities, cultural stigma around mental health, and disparities in health financing and service availability, that accumulate over the lifespan. Targeting these broader determinants through systemic interventions may yield more sustainable improvements in mental health outcomes than relying solely on individualized care models.

Limitations: Use of a self-report questionnaire rather than a clinical interview should also be considered a limitation. Furthermore, it should be pointed out that this assessment serves only as a screening for MDD, but does not allow for a diagnosis

Response

Thank you for this insightful comment. We agree that much as the MINI is a widely used and validated structured diagnostic interview, it is not a substitute for a full clinical evaluation by a mental health professional. As suggested, we now acknowledged this limitation in the manuscript. Specifically, we have added the following sentence to the limitation section (pages 18, lines 371 – 375):

"Additionally, while the MINI is a widely recognized tool for assessing MDD and provides a standardized criteria based on the diagnostic and statistical manual of mental disorders—fifth edition (DMS-5), it may not capture all culturally nuanced manifestations of depression and its reliance on participants' responses poses risk of misclassfications—it is not a substitute for full clinical diagnosis by a mental health professional."

Methods

How were the individuals who were assessed for MDD selected from the pool of control participants?

Response:

All NeuroGAP-Psychosis study's control participants were systematically assessed for MDD with no additional selection process. Because this assessment was integrated into the NeuroGAP-Psychosis protocol partway through the study, a total of 7,073 NeuroGAP-Psychosis study control participants were assessed for MDD out of the total number of 21,238 controls. We have now highlighted this in the revised manuscript and we have additionally outlined the exclusion criteria for NeuroGAP-Psychosis study control participants (page 19, lines 414 – 425):

"The exclusion criteria for NeuroGAP-Psychosis study's control participants were: 1) having a psychotic disorder diagnosis or history of psychotic symptoms, 2) currently taking psychiatric medication for psychosis, 3) acute levels of alcohol or substance abuse as demonstrated by being a current inpatient or under acute medical care for these disorders, not fluent in one of the languages the consent form was translated into and not being able to consent to the study as defined by the University of California, San Diego Brief Assessment of Capacity to Consent questionnaire (UBACC).[49] All NeuroGAP-Psychosis study's control participants were systematically assessed for current and lifetime MDD using the MINI [20] with no additional selection process and are included in this analysis. Because assessment for MDD was integrated into the NeuroGAP-Psychosis study protocol partway through the study, only 7,073 out of the total 21,238 control participants were assessed for MDD. The NeuroGAP-Psychosis …"

Clinical investigations: Please provide information on the reliability and validity of the tools in the study countries.

Response:

We have provided information on the reliability of the MINI (Cronbach's alpha = 0.8, page 20, line 0.8). We could not determine the validity of the tool due to lack of data. We have however highlighted lack of cultural adaptation and validation of the assessment tool as a limitation of this study (limitations section, page 18, lines 371 – 376:

"Additionally, while the MINI is a widely recognized tool for assessing MDD and provides a standardized criteria based on the diagnostic and statistical manual of mental disorders—fifth edition (DSM-5), it may not capture all culturally nuanced manifestations of depression and its reliance on participants' responses poses risk of misclassfications—it is not a substitute for full clinical diagnosis by a mental health professional. Future studies should endeavour to validate assessment tools for MDD for their use in SSA."

Ethical considerations
Please describe referral procedures for participants who screened positive for MDD.

Response:

Participants who screened positive on the MINI were referred to a mental health specialist for further assessment and management. This has been included in the revised manuscript (page 20, lines 452 - 453):

"Participants who screened positive on the MINI were referred to a mental health specialist for further assessment and management."

Data analyses
I was wondering about the appropriateness of running a multilevel model with only four level 2 units (countries). In my opinion including country as fixed effect (dummy variable) would be more appropriate with such a small number of level 2 units.

Response:

Thank you for this important observation. We opted to use multilevel logistic regression to explicitly account for the hierarchical structure of the data, and crucially to estimate the intraclass coefficients (ICCs), which quantify the proportion of variance attributable to between-country differences. We acknowledge that estimating random effects with a small number of levels can be less stable. However, there is a conceptual difference between including country as a fixed-effect covariate and using a multilevel model. The multilevel model explicitly accounts for dependencies between observations nested within the same country, while the fixed-effect specification does not. Our primary interest in the multilevel model was to quantify variance partitioning through ICCs — which is not possible in fixed-effects models, as they do not estimate between-group variance. To address concerns about model robustness, we conducted a sensitivity analysis using a fixed-effects specification (with countries entered as dummy variables), and the substantive findings remained consistent. These results are now mentioned in the manuscript.

This has been explained in the section under data analysis (pages 22-23, lines 492 – 517) which has been slightly revised:

"To examine factors associated with MDD, we employed a multilevel logistic regression model to account for the hierarchical structure of the data, with individuals nested within four different countries, while estimating the intraclass correlation coefficients (ICCs). The ICCs reflect the proportion of variance attributable to between-country differences. Factors such as age, sex, level of education, alcohol use and comorbid physical health conditions (e.g. high blood pressure) which have previously been associated with MDD particularly in SSA, were selected for analysis. Conceptionally, inclusion was guided by the biopsychosocial model of depression which integrates biological, psychological and social determinants of depression.

Model selection was guided by the likelihood ratio test (LRT) to ensure that only variables that significantly improved model fit were retained. The modeling process followed a sequential approach. First, model 1 included key socio-demographic variables (age, sex, education level, living arrangement, and marital status), selected a priori based on theoretical and empirical evidence. Model 2 extended model 1 by incorporating individual-level behavioral and clinical characteristics, including alcohol use, substance use, history of traumatic life events, and current physical illness. Variables were retained only if their inclusion significantly improved model fit based on LRT. Finally, model 3 introduced country-level characteristics, such as geographical location, to assess their influence while controlling for individual-level factors.

The intraclass correlation coefficient (ICC) was calculated for each model to quantify the proportion of total variance attributable to between-country differences. A variable was considered to be meaningfully associated with MDD only if it remained statistically significant in the final model and contributed to model fit as determined by LRT. To assess the robustness of the findings, a sensitivity analysis was performed by systematically excluding individual countries and re-estimating the models. A two-sided p-value <0.05 was considered statistically significant."

I would also expect a description of the testing of statistical assumptions of regression analysis.

Response

Thank you for highlighting this. We indeed tested key assumptions underlying logistic regression analyses. These have now been provided under the last paragraph of the statistical analysis section (page 23, lines 518 – 525):

"We assessed key statistical assumptions to ensure model validity. Multicollinearity was evaluated using variance inflation factors, and all values were within acceptable limits (<10), indicating no strong correlations among predictor variables. For continuous variables such as age, body mass index, and K10 scores, we assessed the linearity of the logistic regression model using visual plots and confirmed that the assumption was reasonably met. Intra-class correlation coefficients (ICCs) were calculated to account for clustering at the country level, and the normality of random effects was evaluated using quantile-quantile (Q-Q) plots. The final model specification was informed by likelihood ratio tests for variable selection."

Reviewer #2 (Remarks to the Author):

The authors present a clear description of the prevalence and factors associated with major depressive disorder in four countries of Africa, a region in which limited epidemiological data has previously indicated alarming rates of depression and in which treatment availability is low. While this is an important area of research conducted in a large and diverse population, I have two significant concerns related to the methodology that greatly diminish the impact of this work.

For one, there is not a clear description of the measures used. Were they adapted at all? What languages were administered in each location? What is the evidence for validity in these populations and what was the reliability in the current sample? While only touched on in the discussion, it seems linguistic understanding and cultural relevance may have been a major issue. Such huge MDD prevalence differences across countries suggest that the MINI may not have worked the same across contexts.

Response:

Thank you for these critical observations. We appreciate the opportunity to clarify the procedures related to the adaptation, administration, and evaluation of the depression assessment tool used in this study. We recognize the importance of cultural and linguistic considerations in psychiatric epidemiology and agree that such factors may influence the diagnostic performance of tools like the MINI across diverse African contexts.

We have highlighted in the methods section why the MINI was used while acknowledging limitation of Western derived tools. We also now report the internal consistency of the tool in the revised manuscript (page 20, lines 444 – 453):

“The MINI was selected due to its brevity, diagnostic alignment with the DSM-5 diagnostic criteria and prior use in SSA. While the MINI was translated into local languages at the different sites and adapted as per the WHO guidelines, we recognize that such tools, originally developed in Western settings may not fully capture local idioms of distress or the unique ways people in different cultures express symptoms. We however found the MINI to have a good internal reliability across the study countries (Cronbach’s alpha = 0.8). Nevertheless, the MINI’s cross-country use enabled diagnostic harmonization and comparability across the study countries. Participants who screened positive on the MINI were referred to a mental health specialist for further assessment and management.”

We also acknowledge in the limitations section that the MINI may not capture all the culturally nuanced manifestations of MDD (page 18, lines 371 – 376):

“Additionally, while the MINI is a widely recognized tool for assessing MDD and provides a standardized criteria based on the diagnostic and statistical manual of mental disorders—fifth edition (DSM-5), it may not capture all culturally nuanced manifestations of depression and its reliance on participants’ responses poses risk of misclassfications—it is not a substitute for full clinical diagnosis by a mental health professional.” Future studies should endeavour to validate assessment tools for MDD for their use in SSA.

Additionally, this sample is not representative of the general population of these countries. Hospital staff are more educated and likely have greater awareness of depression, and people seeking care also likely have a physical health issue, which here and in other studies is associated with depression. Were the caretakers those of people with psychosis? That too would likely bias prevalence.

Response:

We acknowledge this observation regarding the potential lack of generalizability of our findings. Indeed, our sample comprised hospital staff, caretakers and individuals visiting general hospital clinics, who may differ from general populations in terms of their levels of education, mental health awareness and physical health status. Although caretakers were not specifically for individuals with psychosis but rather patients attending various medical clinics, this represents a subgroup with potentially different risk profiles for MDD. We have strengthened this observation in the revised manuscript under the limitation section (page 18, lines 371 – 392):

“Additionally, while the MINI is a widely recognized tool for assessing MDD and provides a standardized criteria based on the diagnostic and statistical manual of mental disorders—fifth edition (DSM-5), it may not capture all culturally nuanced manifestations of depression and its reliance on participants’ responses poses risk of misclassfications—it is not a substitute for full clinical diagnosis by a mental health professional. Also, given the selection criteria for the participants from the hospital settings and included hospital staff, caretakers of general medical patients and patients attending various medical clinics, our study participants may not truly represent a general population. The subgroups may differ from the general population regarding education level, awareness of mental health issues and possibly different socioeconomic profiles. Additionally, given that participation was volunteer based,

we may not have captured the people most severely affected by MDD hence the likelihood of underrepresentation of the most disadvantaged people in the communities studies. Also, since the diagnostic tool used was developed in Western contexts, this could be less sensitive to the cultural expression of MDD in African populations hence leading to potential misdiagnosis. Future multi-country population-based studies are needed to better generalize findings across broader populations. Also, due to lack of data, we did not examine the effect of socioeconomic status on the observed association, despite socioeconomic factros being widely recognized as important determinants of mental health , including MDD. Future studies should prioritize collection of this data to better understand how social determinants interact with clinical and demographic factors in shaping MDD outcomes in Africa."

Considering the much lower prevalence of MDD identified in this study compared to others conducted in the region, I am concerned these issues have significantly impacted the results and made difficult their interpretation.

Response:

Thank you for pointing out this important concern. We agree that the lower prevalence rates identified in our study compared to previously reported regional estimates likely reflect methodological differences and specific aspects of our sample selection. We have highlighted this as a limitation to our study (Please refer to the response in the comment above).

A few more minor comments below:

The abstract should note the much lower prevalence identified here than in prior research and a potential reasoning for why.

Response:

Thank you for this helpful suggestion. We have revised the abstract to highlight this. However, we have also made other revisions to the abstract in order to keep it to the maximum word count of 150 which is required by the journal (page 3):

"Major depressive disorder (MDD) significantly contributes to the mental health burden in Africa, requiring insights into prevalence and risk factors to inform culturally responsive care. This study assessed current and lifetime MDD prevalence and associated factors among 7,073 adults from hospital settings in Uganda, Kenya, Ethiopia, and South Africa within the NeuroGAP-Psychosis study. Prevalence was 0.9% for current MDD and 5.1% for lifetime MDD, substantially lower than prior estimates from the region. Lower rates may be due to the population studied or cultural variations in expression of symptoms. Multilevel logistic regression identified significant associations between current MDD and negative life events, alcohol use, and Kessler psychological distress scores, while lifetime MDD was linked to age, female sex, chronic pain, frequent headaches, and positive psychosis screening. These findings underscore the need for targeted mental health interventions tailored to the identified factors to reduce the burden of MDD in diverse African contexts."

The association of sex and current MDD is inversed. Males are significantly LESS likely to have current MDD. The way it is written now (line 140) suggests they have higher odds.

<u>Response:</u>

Thank you for this observation. We have revised this sentence (now line 155 - 156, page 7):

"In the multilevel models, male sex at birth was marginally significantly associated with lower odds of current MDD (aOR = 0.47 [95% CI: 0.22; 1.02])."

The first mention of the Eastern and Southern African categories (line 153) should indicate which country was included in each category (ex. Uganda could be considered both).

<u>Response:</u>

Thank you for this helpful suggestion, we have updated the manuscript to clarify which countries were included in the Eastern and Southern Africa categories at first mention (page 8, lines 168 - 169):

"The geographical location, represented by living in Eastern Africa (Uganda, Kenya and Ethiopia) compared to Southern Africa (South Africa), ……"

Typo: Line 108, 167, 207, 246, 387

<u>Response:</u>

Thank you for pointing this out. We have carefully reviewed and corrected the typos in lines 108, 167, 207, 246 and 387 now lines 112, 182, 229, 270 and 455 respectively.

Yours sincerely,

Dr Allan Kalungi, Ph.D., AFHEA
Corresponding author.
